# Human sensory-like neuron cultivation—An optimized protocol

**DOI:** 10.3389/fnins.2024.1429694

**Published:** 2024-10-03

**Authors:** Nicole Michelle Schottmann, Julia Grüner, Frederik Bär, Franziska Karl-Schöller, Sabrina Oerter, Nurcan Üçeyler

**Affiliations:** ^1^Department of Neurology, University Hospital Würzburg, Würzburg, Germany; ^2^Institute of Tissue Engineering and Regenerative Medicine, University Hospital Würzburg, Würzburg, Germany

**Keywords:** induced pluripotent stem cells, induced sensory-like neurons, mixed cell cultures, differentiation protocol, *in vitro* model, multi-electrode array

## Abstract

**Introduction:**

Reprogramming of human-induced pluripotent stem cells (iPSCs) and their differentiation into specific cell types, such as induced sensory-like neurons (iSNs), are critical for disease modeling and drug testing. However, the variability of cell populations challenges reliability and reproducibility. While various protocols for iSN differentiation exist, the development of non-iSN cells in these cultures remains an issue. Therefore, standardization of protocols is essential. This study aimed to improve iSN culture conditions by reducing the number of non-iSN cells while preserving the survival and quality of iSNs.

**Methods:**

iSNs were differentiated from a healthy control iPSC line using an established protocol. Interventions for protocol optimization included floxuridine (FdU) or 1-β-D-arabinofuranosyl-cytosine hydrochloride (AraC) treatment, magnetic-activated cell sorting (MACS), early cell passaging, and replating. Cell viability and iSN-to-total-cell-count ratio were assessed using a luminescent assay and immunocytochemistry, respectively.

**Results:**

Passaging of cells during differentiation did not increase the iSN-to-total-cell-count ratio, and MACS of immature iSNs led to neuronal blebbing and reduced the iSN-to-total-cell-count ratio. Treatment with high concentrations and prolonged incubation of FdU or AraC resulted in excessive cell death. However, treatment with 10 μM FdU for 24 h post-differentiation showed the most selective targeting of non-iSN cells, leading to an increase in the iSN-to-total-cell count ratio without compromising the viability or functionality of the iSN population. Replating of iSNs shortly after seeding also helped to reduce non-iSN cells.

**Conclusion:**

In direct comparison with other methods, treatment with 10 μM FdU for 24 h after differentiation shows promise for improving iSN culture purity, which could benefit downstream applications in disease modeling and drug discovery. However, further investigations involving multiple iPSC lines and optimization of protocol parameters are warranted to fully exploit the potential of this method and enhance its reproducibility and applicability. Overall, this study provides valuable insights into optimizing culture conditions for iSN differentiation and highlights the importance of standardized protocols in iPSC-based research.

## 1 Introduction

The generation of human-induced pluripotent stem cells (iPSCs; Takahashi and Yamanaka, 2006) and their differentiation into specific target cells, such as induced sensory-like neurons (iSNs; Chambers et al., 2009) has emerged as a potent method for disease modeling and drug testing. Standardization of methodological procedures is crucial to reduce technical variability to a minimum and to ensure reliability and reproducibility (Lampert et al., 2020; Volpato and Webber, 2020). Currently, there are two protocols for differentiating iSNs: one based on combined small-molecule inhibition (Chambers et al., 2012) and the other involving the overexpression of transcription factors (Blanchard et al., 2015). Additionally, commercially available options include RealDRG™, and the accelerated Senso-MM™ protocol, both from Anatomic Incorporated (Walsh et al., 2020; Kalia et al., 2024). Application of the small-molecule protocol also results in the generation of non-iSN cells with high variability in morphology and counts between differentiations (Schwartzentruber et al., 2018). This cellular heterogeneity challenges correct data allocation and interpretation.

To increase culture purity, the number of non-neuronal cells can be reduced by the application of cytostatic compounds used in chemotherapy (Hilgenberg and Smith, 2007; Thirumangalakudi et al., 2009; Irobi et al., 2010; Liu et al., 2013; Schwieger et al., 2016; Clark et al., 2021). This inhibits the proliferation of non-iSNs by mainly targeting mitotic cells. However, no standardized protocol is available for the best compounds, time points, and concentrations (Hilgenberg and Smith, 2007; Thirumangalakudi et al., 2009; Irobi et al., 2010; Liu et al., 2013; Schwieger et al., 2016; Clark et al., 2021). Further approaches achieved higher ratios of iSN-to-non-iSN cells by additional splits during the first days of iSN differentiation (Clark et al., 2017) and neuronal cell sorting (Hirano et al., 2021).

We aimed to improve the culture conditions of human iSNs by reducing the number of non-iSN cells in culture while preserving iSN quality. We assessed cell viability and iSN-to-total-cell count ratios after testing defined *in vitro* interventions. Among these, we found that the application of the chemotherapeutic floxuridine (FdU) 10 μM for 24 h after differentiation yielded the best results. We demonstrate that this treatment improves culture purity by being the most selective in targeting non-iSN cells without compromising the long-term viability or functionality of the iSN population. FdU led to an increase in the iSN-to-total-cell count ratio indicating a reduction of non-iSN cells. We further show that these iSNs express sensory neuron-specific marker proteins and are electrically active.

## 2 Materials and methods

### 2.1 iSN differentiation

**Figure 1:**
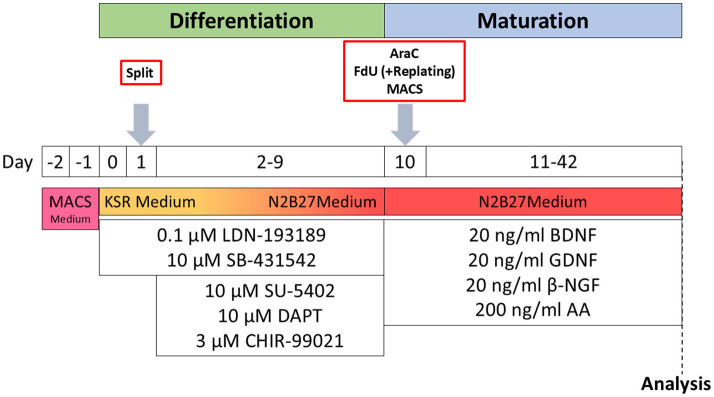
Schematic timeline of iSN differentiation and experimental procedures. AA, ascorbic acid; BDNF, brain-derived neurotrophic factor; βNGF, nerve growth factor beta; GDNF, glial cell line-derived neurotrophic factor; KSR Medium, KnockOut medium; MACS Medium, StemMACS iPS-Brew.

iSNs were differentiated from a healthy control iPSC line as described recently (Klein et al., 2024). In brief, 1.125^*^10^6^ iPSCs were seeded into growth factor-reduced Matrigel-coated wells (bMg, Corning, Corning, NY, USA) in a six-well plate in 2 ml StemMACS iPS-Brew (Miltenyi Biotec, Bergisch Gladbach, Germany). The medium was supplemented with 100 U/ml penicillin/streptomycin (Gibco, Waltham, MA, USA) and 10 μM Y27632 (Miltenyi Biotec, Bergisch Gladbach, Germany) on day −2. On day −1, the StemMACS medium was changed, and differentiation was started on day 0 after passaging. Cells were then cultivated in KnockOut medium [KSR; KnockOut DMEM/F12, 2 mM GlutaMAX, 15% KnockOut Serum Replacement, 100 μM 2-mercaptoethanol, 0.1 mM minimum essential medium non-essential amino acids + 100 U/ml penicillin/streptomycin (all: Thermo Fisher Scientific, Waltham, MA, USA)]. The KnockOut medium was supplemented with a two-inhibitor cocktail (2i) containing 100 nM LDN-193189 (STEMCELL Technologies, Vancouver, Canada) and 10 μM SB-431542 (Miltenyi Biotec, Bergisch Gladbach, Germany). Three inhibitor (3i) cocktail [10 μM SU-5402, 10 μM DAPT (both: Sigma-Aldrich, St. Louis, MO, USA), and 3 μM CHIR-99021 (Axon Medchem, Groningen, Netherlands)] was added from day 2. Starting on day +4, KSR medium was replaced in 25% steps with N2B27 medium [N2; DMEM/F12 GlutaMAX, 1X B-27 Plus Supplement, 1X N-2 Supplement, 100 U/ml penicillin/streptomycin (all: Thermo Fisher Scientific, Waltham, MA, USA)] every other day. The cells were passaged on bMg-coated 12-mm coverslips with a 1:2 ratio on day 10 using TrypLE Express (Thermo Fisher Scientific, Waltham, MA, USA) for 8–20 min. If not stated otherwise, neuronal maturation medium consisted of N2B27 medium supplemented with 20 ng/ml BDNF, 20 ng/ml GDNF, 20 ng/ml NGFb (all: Peprotech, Rocky Hill, NJ, USA), and 200 ng/ml ascorbic acid (Sigma-Aldrich, St. Louis, MO, USA). iSNs were matured for 6 weeks (Figure 1).

### 2.2 FdU treatment

iSNs were seeded on bMg-coated coverslips on day +10 of differentiation and treated with FdU (Santa Cruz Biotechnology, Dallas, TX, USA) at concentrations of 1, 5, 10, and 20 μM for 24, 48, and 72 h. For later time points, half medium change was performed to ensure stable FdU concentrations. The results were compared to a control (ctrl) group treated with 10 μM FdU for 24 h.

### 2.3 1-β-D-arabinofuranosyl-cytosine hydrochloride treatment

iSNs were seeded on bMg-coated coverslips on day +10 of differentiation. Cells were incubated with 0.5, 1, 5, and 10 μM of 1-β-D-arabinofuranosyl-cytosine hydrochloride (AraC, Sigma-Aldrich, St. Louis, MO, USA) over 24, 48, 72, 120, and 168 h. Stable AraC concentrations were ensured by performing half-media changes for longer incubation periods. As a control condition, cells were treated with 10 μM FdU for 24 h.

### 2.4 Magnetic-activated cell sorting

Following a published protocol (Hirano et al., 2021), iSNs were sorted using magnetic-activated cell sorting (MACS) on day +10 of differentiation. Cells were detached, resuspended in 1 ml phosphate-buffered saline with Mg^2+^ and Ca^2+^ (PBS ++, PBS, Merck, Darmstadt, Germany), and counted as described in iSN differentiation. The cells were sorted using Neural Crest Stem Cell MicroBeads, human (Miltenyi Biotec, Bergisch Gladbach, Germany). Afterward, the cells were seeded on bMg-coated coverslips in 1 ml maturation medium, additionally containing 10 μM DAPT. Two further approaches were tested: A cell strainer was used to reduce cell clusters, and a second MACS step was included for higher purification. The cells incubated with 10 μM FdU for 24 h were used as control.

### 2.5 Split on day +2 of differentiation

Cells were split on day +2 of differentiation following Clark et al. (2017). The cells were detached after washing with PBS using 0.5 mM EDTA (Invitrogen, Carlsbad, CA, USA) for 5 min at 37°C. They were then split 1:1 onto bMg-coated wells in 6-well plates containing KSR medium supplemented with 10 μM Y27632 and LDN, SB, CHIR, DAPT, and SU as described in iSN differentiation.

### 2.6 Replating of iSNs

iSNs were replated on freshly bMG-coated coverslips after 10, 15, 20, 25, 30, and 45 min following the day +10 split. Media were directly transferred on a new coverslip without further resuspension. Old coverslips were substituted with fresh media each.

### 2.7 Immunocytochemistry

Cells were fixed using 4% paraformaldehyde (PFA; Electron Microscopy Sciences, Hatfield, USA) for 20 min at room temperature followed by three washing steps using PBS++. Blocking solution was applied for 30 min at room temperature and consisted of 10% fetal bovine serum (Honduras Origin, Sigma-Aldrich, St. Louis, MO, USA) and 0.1% saponin (Sigma-Aldrich, St. Louis, MO, USA) diluted in PBS++. Anti-voltage-gated sodium channel 1.8 (Nav1.8) antibody (1:100, Abcam, Cambridge, UK), peripherin (PRPH) antibody (1:250, AF488/AF647, Santa Cruz Biotechnology, Dallas, TX, USA), and anti-beta-III-tubulin (TUJ1) antibody (1:500, Abcam, Cambridge, UK) diluted in blocking solution were applied overnight at 4°C. Unbound antibody was washed with PBS++ including 4′,6-diamidino-2-phenylindole for initialization (DAPI; 1:10 000, Sigma-Aldrich, St. Louis, MS, USA). Then, secondary antibodies (donkey anti-chicken AF488 and donkey anti-rabbit Cy3; Jackson ImmunoResearch Europe, Cambridge House, St. Thomas' Place, Ely, UK) were applied for 30 min after washing primary antibodies three times with PBS++ and before adding DAPI. Cells were mounted in Aqua-Poly/Mount (Polysciences, Warrington, PA, USA). Photomicrographs for further analysis were taken with an inverted fluorescence microscope (Leica DMi 8, Leica Microsystems, Wetzlar, Germany). The analysis was performed using a CellProfiler 4.1.3 Ink pipeline. Other images were captured using a Zeiss Axio Imager M2 microscope.

### 2.8 Cell viability assay

ISNs were seeded on bMg-coated 96-well plates (Greiner Bio-One GmbH, Frickenhausen, Germany) on day +10 of differentiation and incubated with AraC or FdU as described above. After 24, 48, and 72 h, a CellTiter-Glo^®^ Luminescent Cell Viability Assay (Promega, Madison, WI, USA) was performed. Cells received 1:1 maturation medium and CellTiter-Glo^®^ Reagent (Promega, Madison, WI, USA). The plates were shaken at 480 rpm for 2 min followed by 10 min incubation at room temperature in the dark. Luminescence was measured using a Tecan Spark multiplate reader (Tecan Trading AG, Männedorf, Switzerland). 10 μM of FdU for 24 h was used as ctrl, while 10% SDS posed as negative ctrl.

### 2.9 Multi-electrode array

The electrical activity of iSNs was assessed using a multiwell multi-electrode array (MEA) system comprising 24 individual wells with 12 electrodes per well (Multi Channel Systems MCS GmbH, Reutlingen, Germany). ISNs were seeded into bMg-coated 24-well plates with PEDOT electrodes on glass (Multi Channel Systems, Reutlingen, Germany) on day +10 of differentiation in neuronal maturation medium supplemented with 10 μM FdU. MEA measurements were performed after 4, 5, and 6 weeks of maturation. Each recording was conducted in a neural maturation medium at 37°C using an internal headstage heating platform for 2 min. The data of all 288 electrodes were captured at a sampling rate of 20 kHz, employing a second-order Butterworth filter with a high-pass cutoff at 300 Hz and a low-pass cutoff at 3,500 Hz. The analysis was conducted using Multiwell-Analyzer software (Multi Channel Systems MCS GmbH, Reutlingen, Germany) with the noise threshold for spike detection set at ±4.5 standard deviations.

### 2.10 Statistics

For statistical analysis and data representation as graphs, SPSS Statistics 27 (IBM, Armonk, NY, USA) and GraphPad PRISM Version 9.5 (GraphPad Software, Inc., La Jolla, CA, USA) were used, respectively. Unless stated otherwise, the data were normalized to the median of the ctrl condition for each differentiation to account for differences between differentiations. Data sets were compared using the Kruskal–Wallis test with Dunn's multiple comparisons for correction or one-way ANOVA with *post-hoc* Tukey's HSD. If necessary, an unpaired *t*-test was performed. Appropriate parametric or non-parametric tests are stated in the respective figure legends of data sets. All data sets include *n* = 3 individual differentiations.

## 3 Results

### 3.1 Passaging of cells during differentiation does not increase iSN-to-total-cell-count ratio

**Figure 2:**
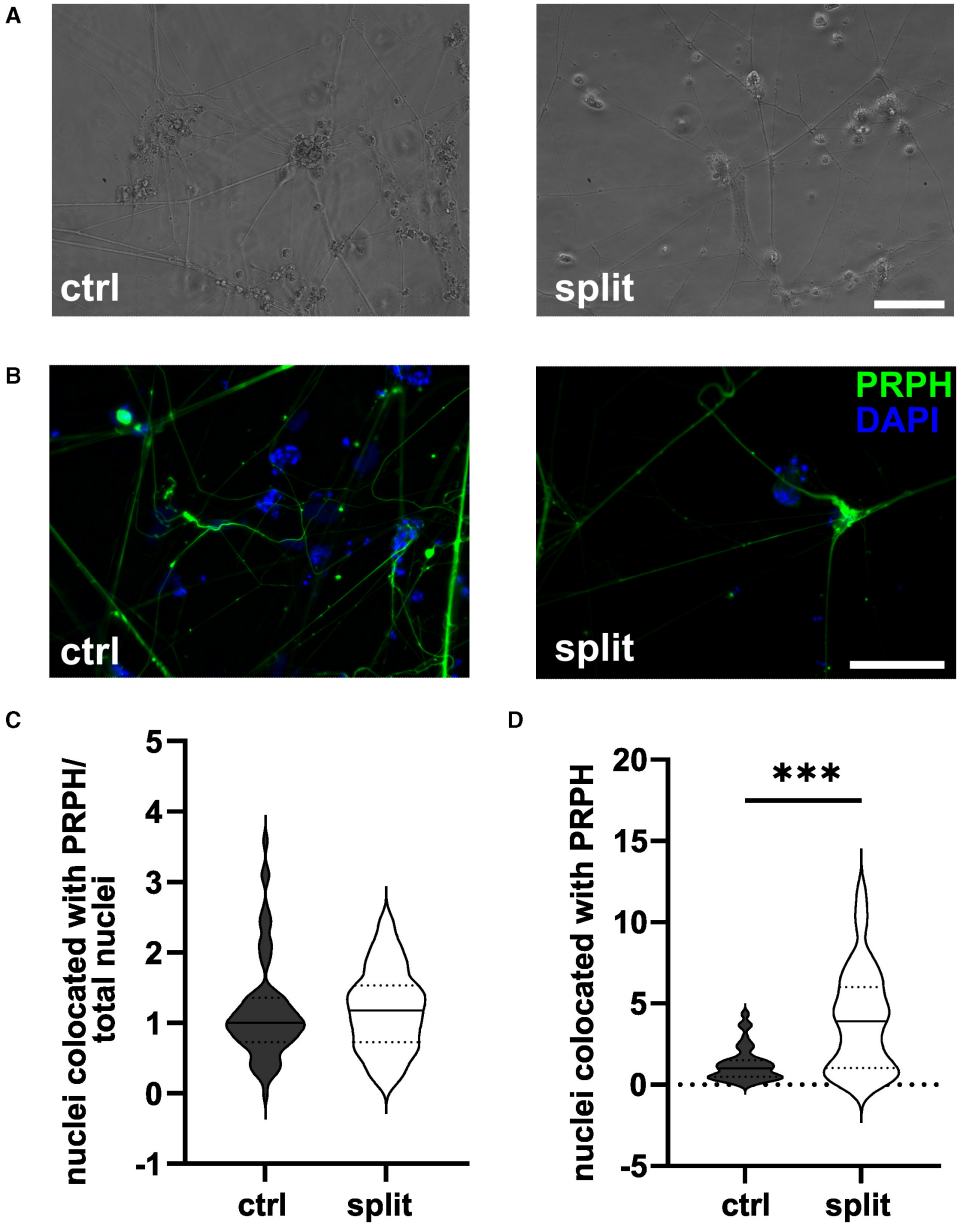
iSNs split on day +2 of differentiation. Representative bright field **(A)** and ICC photomicrographs **(B)** of split cells compared to the ctrl. While the ratio of the iSN-to-total-cell-count remained similar between the split and ctrl cells **(C)**, the total number of cells produced could be improved by split on day +2 of differentiation **(D)**. The data of n = 3 differentiations are presented as violin plots and analyzed using an unpaired *t*-test. ***p < 0.001. ctrl, control; ICC, immunocytochemistry; PRPH, peripherin; scale bar = 100 μm.

Passaging of cells on day +2 of differentiation did not result in morphological changes as shown in representative bright field (Figure 2A) and ICC photomicrographs (Figure 2B). Analysis of ICC images via a cell profiler pipeline did not reveal any differences in the iSN-to-total-cell-count ratio between cells split on day +2 of differentiation and ctrl (Figure 2C). While the ratio of iSN-to-non-iSN cells did not change, the total number of generated cells increased significantly (*p* < 0.001; Figure 2D) when comparing the iSN count after the split (1.0 ± 0–4.4) to the iSN count in the ctrl condition (3.9 ± 0.08–12.1).

### 3.2 Sorting of cells on day +10 of differentiation does not increase the ratio of iSN-to-total-cell-count

**Figure 3:**
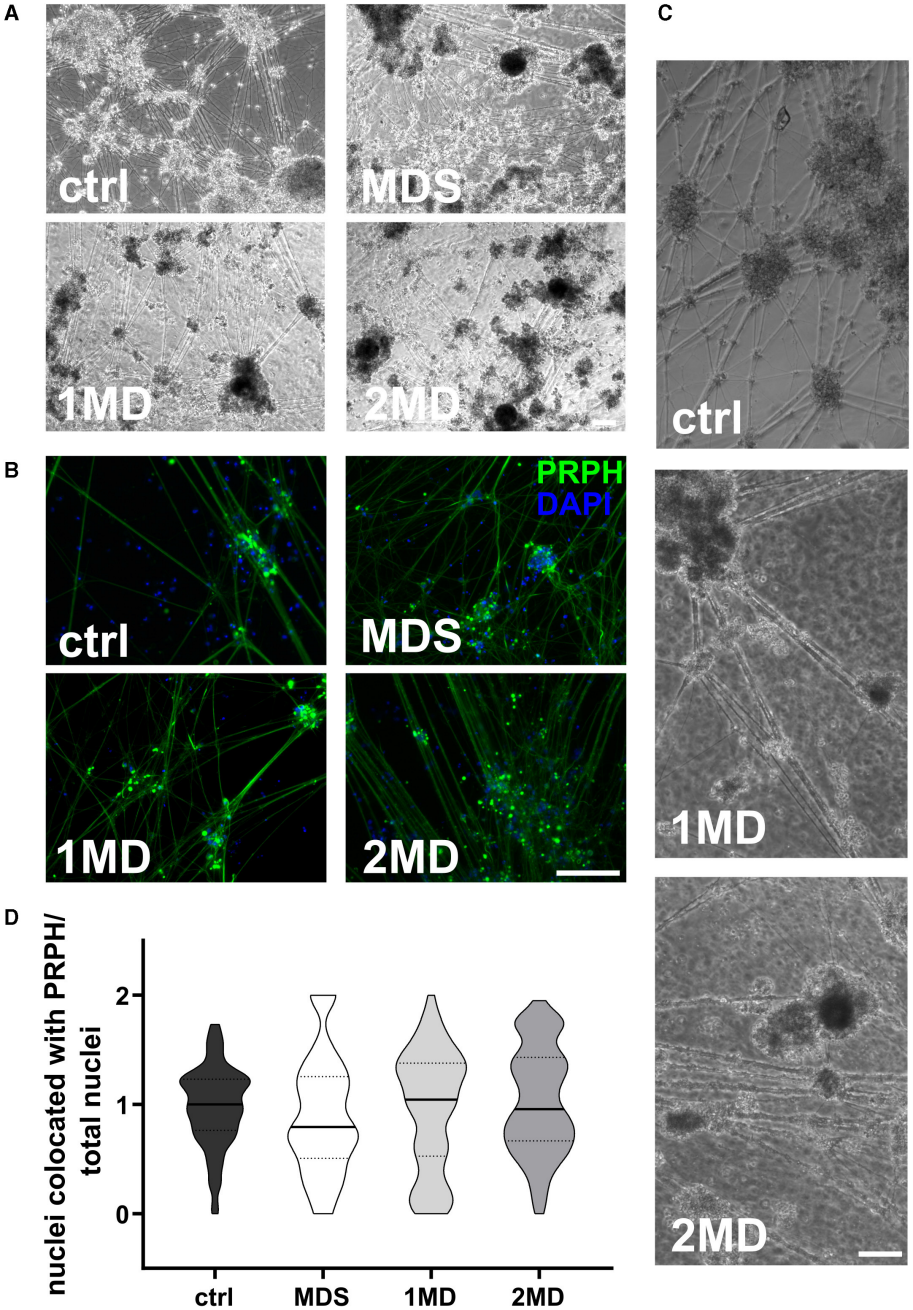
Sorting of iSNs on day +10 of differentiation. Representative bright field microscopy **(A)** and ICC photomicrographs **(B)** show increased neuronal blebbing **(C)** of iSNs after MACS treatment. Furthermore, non-neuronal cell types changed to a more homogeneous morphology. **(D)** MACS applications resulted in an iSN-to-total-cell-count-ratio comparable to ctrl. The data of *n* = 3 differentiations are presented as violin plots and analyzed using the Kruskal–Wallis test with Dunn's multiple comparisons. ctrl, control; ICC, immunocytochemistry; MD, MACS and DAPT treatment; MDS, MACS and DAPT treatment combined with cell strainer; PRPH, peripherin. Scale bar = 100 μm.

MACS of immature iSNs on day +10 of differentiation led to neuronal blebbing (Figures 3A–C). Comparison of defined MACS strategies did not show a difference in iSN-to-total-cell-count ratio compared to ctrl (Figure 3D). In all cases, the application of MACS led to a reduced number of iSNs and flow-through mostly containing other cell types instead of iSNs.

### 3.3 High concentrations and long FdU or AraC incubation result in excessive cell death

**Figure 4:**
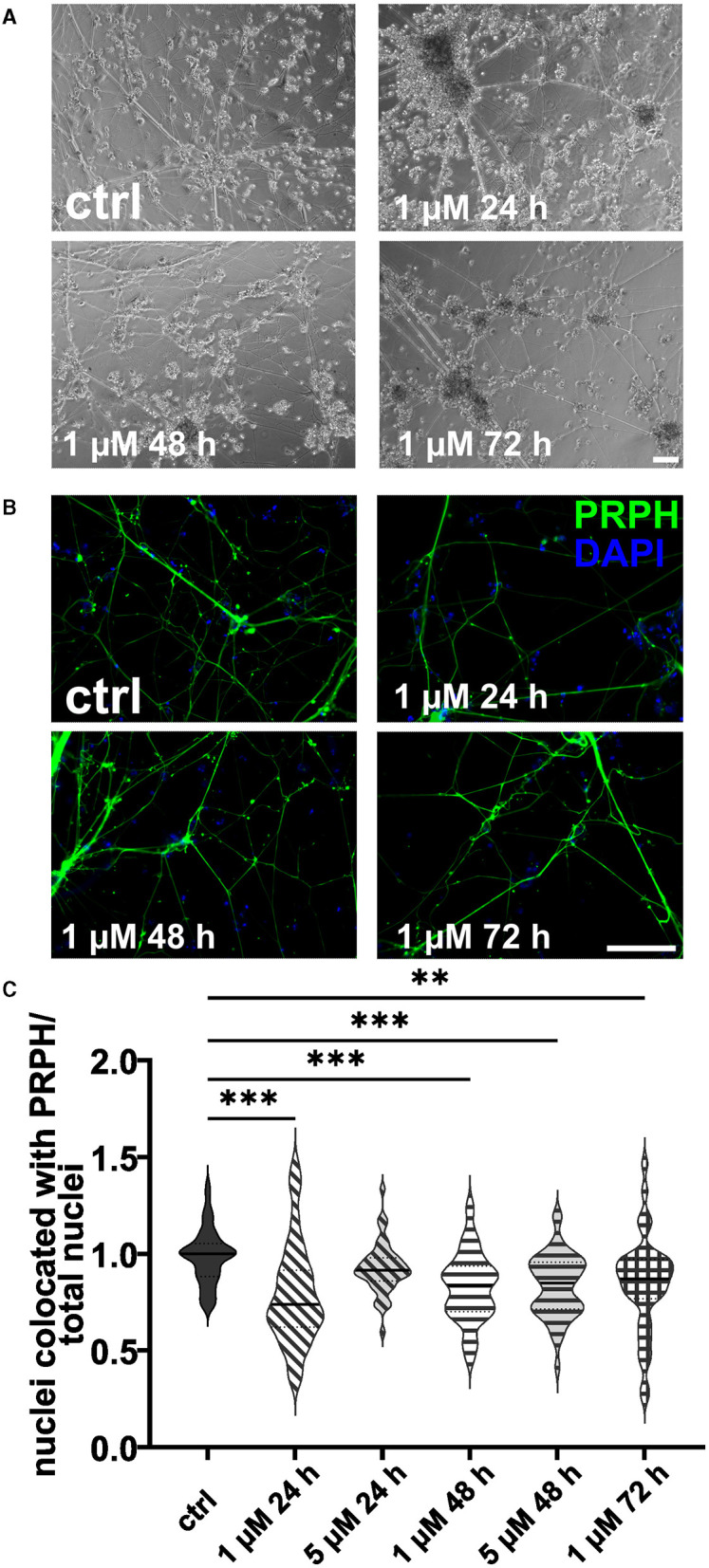
iSNs treated with FdU on day +10 of differentiation. Bright field microscopy **(A)** and ICC photomicrographs **(B)** did not show an improvement of iSN morphology and reduced number of other cell types with 1 μM FdU over distinct time points (24, 48, and 72 h) compared to the ctrl. Most conditions in which cells survived showed a smaller iSN-to-total-cell-count ratio than the ctrl. Application of 5 μM FdU for 24 h results in an iSN-to-total-cell-count ratio close to the ctrl **(C)**. The data of *n* = 3 differentiations are presented as violin plots and analyzed using the Kruskal–Wallis test with Dunn's multiple comparisons. **p < 0.01; ***p < 0.001. ctrl, control; ICC, immunocytochemistry; PRPH, peripherin. Scale bar = 100 μm.

ISNs treated with 20 μM FdU did not survive, independent of the duration of incubation. This was also observed when using 10 μM FdU for more than 48 h. While incubation with 5 μM FdU for more than 72 h did not result in complete cell death, only a few iSNs survived, showing morphological signs of stress, such as neuronal blebbing (Figures 4A, B). Therefore, experiments were only repeated for lower concentrations. Application of 1 μM FdU for 24 h was not as effective as the control condition in reducing non-iSN cells (Figure 4C): Also with a longer incubation time of 48 and 72 h, iSN-to-total-cell-count ratio remained smaller (*p* < 0.01) than the ctrl. Incubation with 5 μM FdU for more than 48 h did not increase culture purity (*p* < 0.001). Only 5 μM applied for 24 h resulted in an iSN-to-total-cell-count ratio similarly comparable to ctrl, but trending to a smaller ratio (Figure 4C), microscopic observation revealed a more pronounced non-iSN layer in most wells.

**Figure 5:**
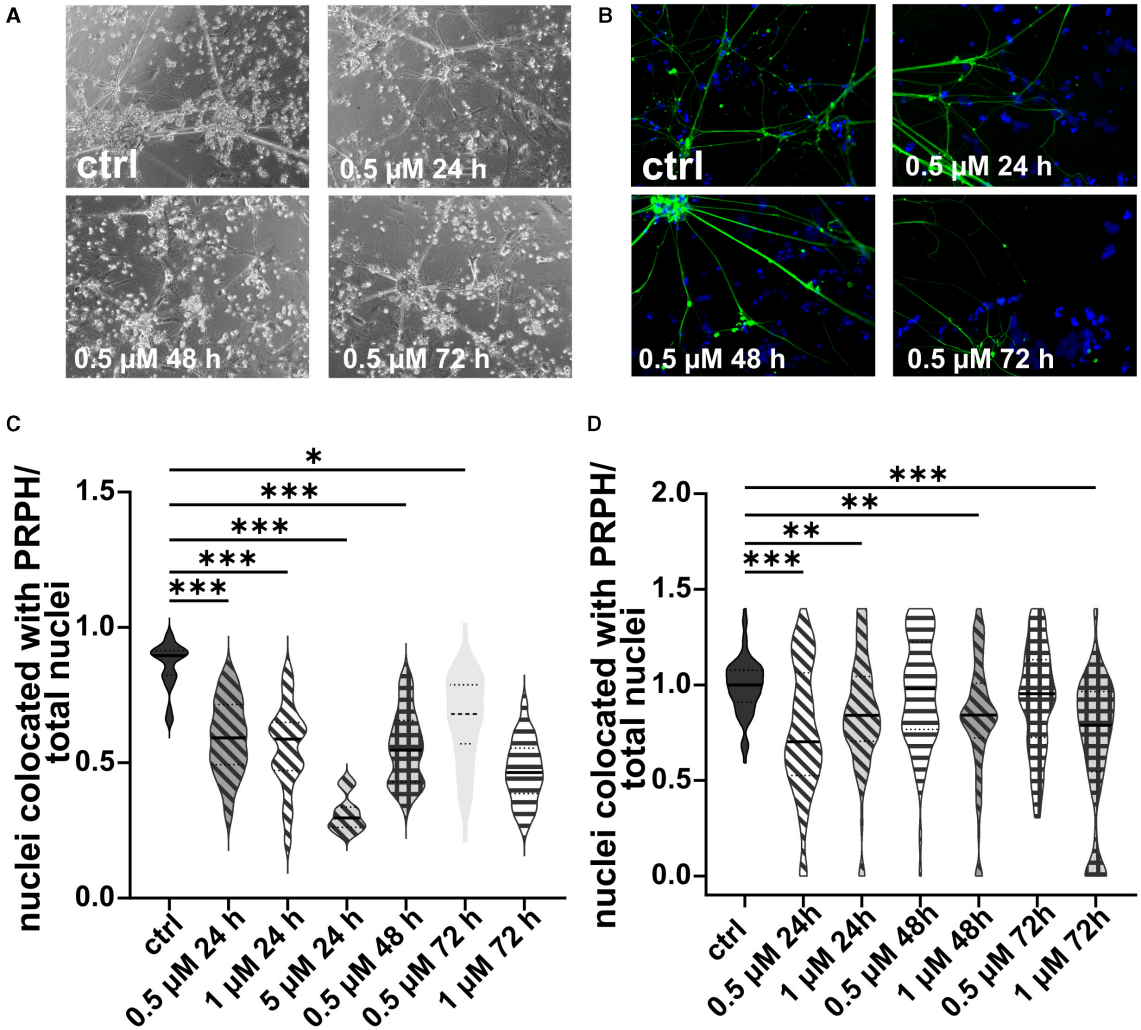
iSNs treated with AraC on day +10 of differentiation. Treatment with 0.5 μM AraC as shown in bright field microscopy **(A)** and ICC photomicrographs **(B)** did not improve iSN morphology and the number of other cell types regardless of incubation time. As most iSNs treated with higher doses and longer treatment periods died, the following conditions could not be integrated into the results: 1 μM for 120 and 168 h, 5 μM for 72, 120, and 168 h; 10 μM for 24, 72, 120, and 168 h. Therefore, after two pilot experiments summarized in **(C)**, the data were not normalized, treatment conditions were adjusted, and we focused only on lower conditions. The ISN-to-total-cell-count ratio was smaller for most conditions where cells survived in comparison with ctrl. Only 0.5 μM AraC for 48 h and 72 h showed similar efficacy as ctrl **(D)**. The data of *n* = 3 differentiations are presented as violin plots and analyzed using the Kruskal–Wallis test with Dunn's multiple comparisons. *p < 0.05; **p < 0.01; ***p < 0.001. ctrl, control; ICC, immunocytochemistry; PRPH, peripherin. Scale bar = 100 μm.

In analogy, excessive cell death was observed in iSNs treated with 5 or 10 μM AraC independent of the incubation time. In contrast, after application of 1 μM AraC for 24 h and 72 h, neuronal cell survival increased. However, after longer incubation times of more than 72 h, the iSNs completely died. Hence, experimental conditions were adjusted to a lower concentration and a shorter incubation time. There was no positive effect on iSN morphology (Figures 5A, B) and counts of non-iSN cells when treated with 0.5 μM AraC for all time points. The ISN-to-total-cell-count ratio decreased significantly (*p* < 0.01) for most treatment conditions where cells survived compared to the ctrl, except for 0.5 μM AraC at 48 and 72 h, which showed similar efficacy to the ctrl (Figures 5C, D). However, we observed that iSNs treated with AraC tended to detach more often.

### 3.4 Replating iSNs shortly after seeding helps reduce non-iSN cells

**Figure 6:**
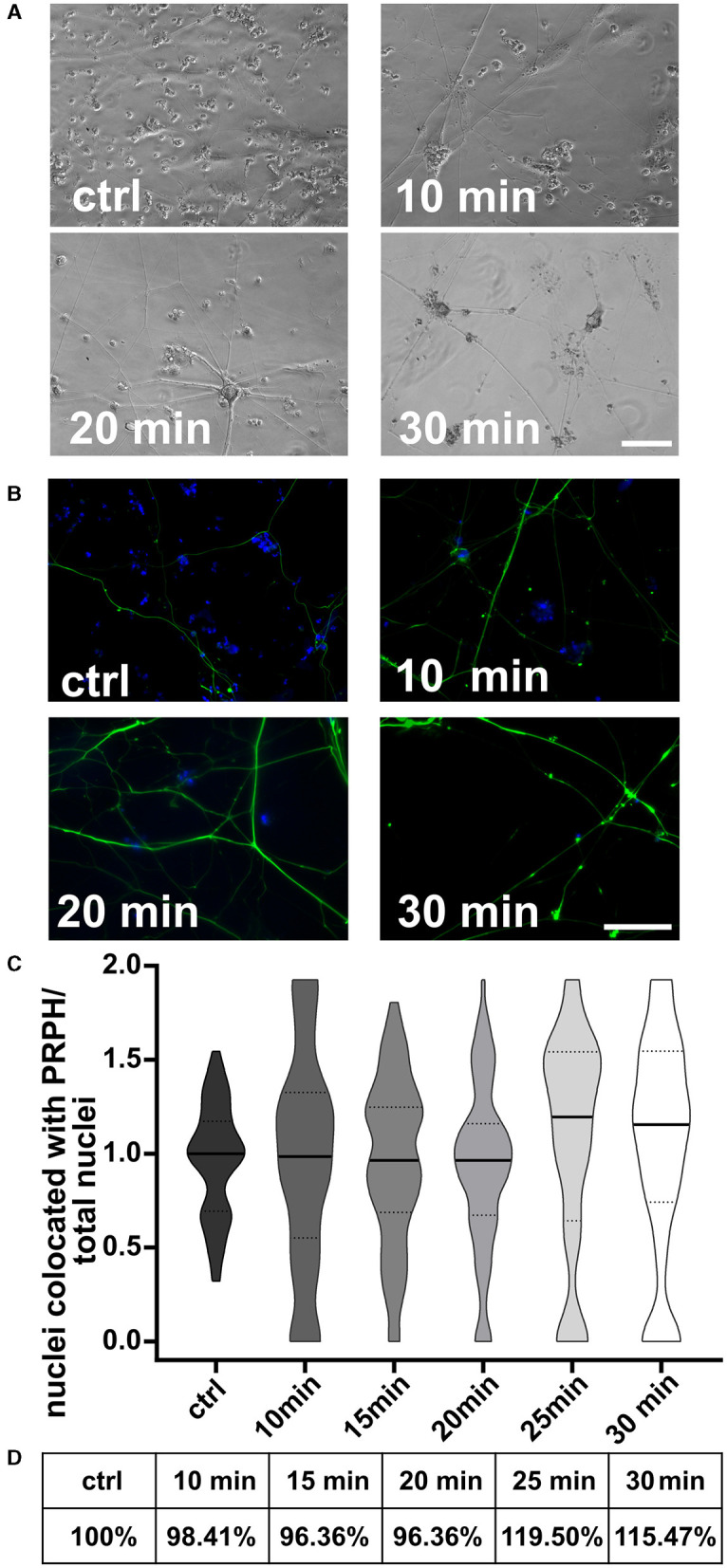
iSNs replated onto new coverslips after day 10 split. Number of non-iSN cells seen in representative bright field microscopy **(A)** and ICC photomicrographs **(B)** of cells replated 10, 20, and 30 min after the initial split (additional to 10 μM FdU treatment) was reduced. **(C)** Replating cells for 25 min was most effective; however, there was only a slight improvement in the iSN-to-total-cell-count ratio compared to the ctrl. **(D)** Replating improved the iSN-to-total-cell-count ratio between 15.47 and 19.50%. The data of *n* = 3 differentiations are presented as violin plots and analyzed using the Kruskal–Wallis test with Dunn's multiple comparisons. ctrl, control; ICC, immunocytochemistry; PRPH, peripherin. Scale bar = 100 μm.

Cells replated 10, 15, and 20 min after the day +10 split showed no differences in morphology (Figures 6A, B) or in the iSN-to-total-cell-count ratio (Figure 6C). However, replating at 25 or 30 min after the initial seeding indicated a tendency toward improvement in the iSN-to-total-cell-count ratio (Figure 6C). In general, replating helped to advance the iSN-to-total-cell-count ratio up to 119.50% (Figure 6D). Replating was also tested after 45 min, but the cells had already completely attached at this time point.

### 3.5 Long exposure times to FdU and AraC affect cell viability

**Figure 7:**
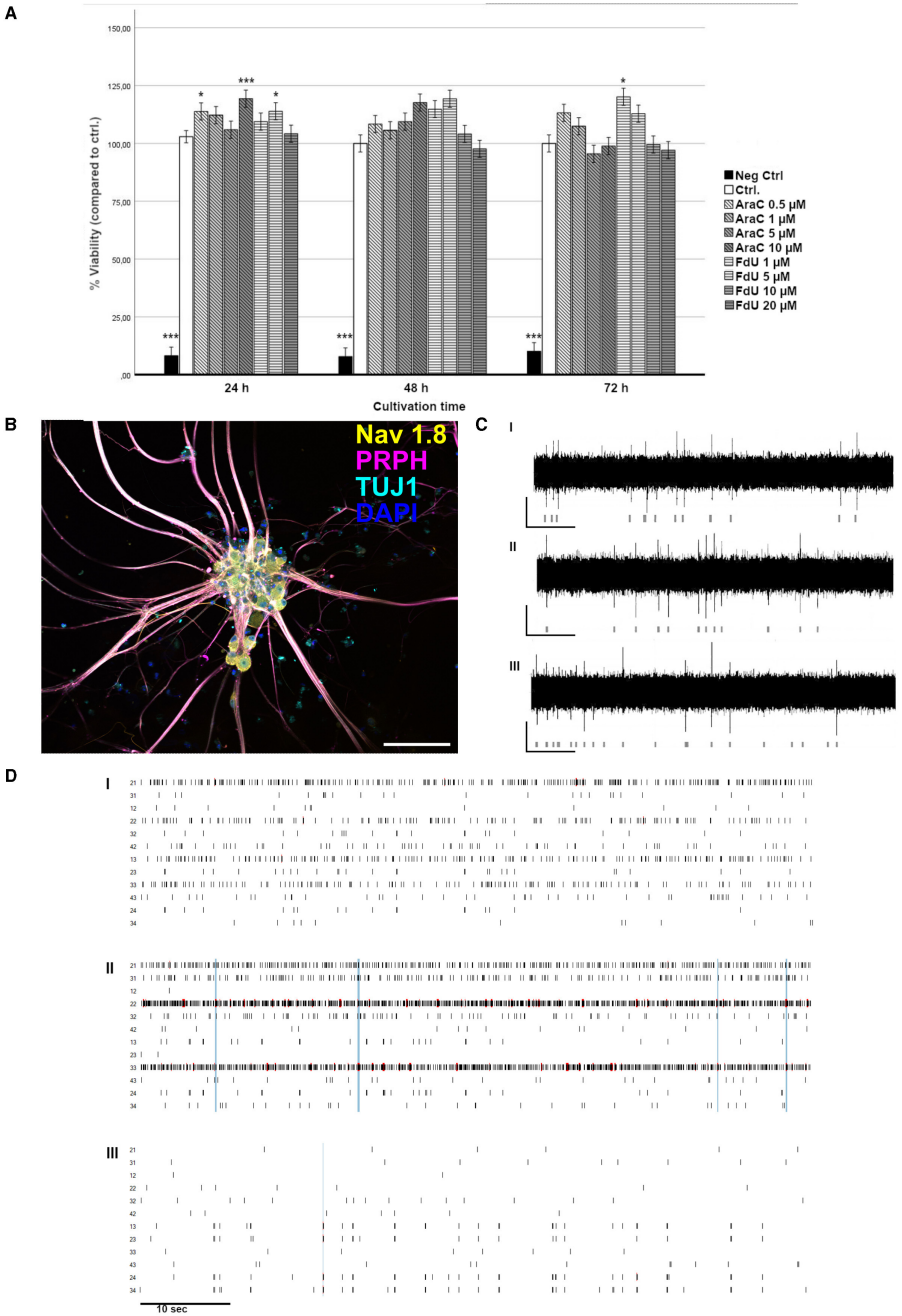
Validation of neuronal integrity. **(A)** Viability assay of iSNs following treatment with increasing concentrations of FdU and AraC for up to 72 h. SDS was used as negative ctrl, 10 μM of FdU for 24 h. The data of *n* = 3 differentiations, one-way ANOVA with *post-hoc* Tukey's HSD. *p < 0.05; ***p < 0.001. **(B)** iSNs generated using our optimized protocol express the sensory neuron marker Nav1.8, the peripheral neuron marker PRPH, and a general neuronal marker TUJ1. The scale bar = 100 μm. **(C, D)** Spontaneous electrical activity in iSNs was measured at 4 (I), 5 (II), and 6 (III) weeks of maturation using a multi-electrode array system, with activity detectable starting from 4 weeks of maturation. Scale: 50 μV/10 s. **(D)** Raster plot of one well over 4 (I), 5 (II), and 6 (III) weeks of maturation showing the single and developing network activity. For **(C, D)** *n* = 3 triplicates from 1 differentiation. ICC, immunocytochemistry; Nav1.8, voltage-gated sodium channel 1.8; PRPH, peripherin; TUJ1, Beta-III-tubulin.

The cell viability was assessed relative to a positive ctrl treated with 10 μM FdU for 24 h (Figure 7A) and SDS as negative ctrl. Treatment of iSNs with 0.5 μM AraC for 24 h resulted in an increase in cell viability (*p* < 0.05). However, extending the exposure to 48 and 72 h at this concentration did not produce relevant changes. Elevating the concentration of AraC to 1 and 5 μM had minimal impact on viability, although a marked reduction was noted after 3 days with 5 μM AraC. While treatment with 10 μM AraC for 24 h initially increased cell viability, this effect diminished by 48 h and declined by 72 h.

For FdU treatments, concentrations of 1 and 5 μM for up to 48 h did not significantly affect viability, except for a slight elevation observed at 72 h with 1 μM FdU and at 24 h with 5 μM FdU. Higher concentrations and longer exposures, particularly 10 μM FdU for 48 and 72 h and 20 μM FdU for 48 and 72 h, resulted in decreased cell viability. No significant changes were detected with 20 μM FdU at 24 h.

### 3.6 iSNs express characteristic markers and are functionally active

The integrity of iSNs cultivated under ctrl conditions was confirmed via expression of the sensory neuron markers Nav1.8, PRPH, and TUJ1 (Figure 7B). To assess the functionality and neuronal activity of our iSNs, we conducted a MEA analysis (Figures 7C, D). The observed background noise could be attributed to several factors, including individual noise from the sensitive reference electrodes, the coating, and residual non-iSN cells in the culture. To mitigate these noise elements, we applied a filter to exclude low-frequency local field potentials, analyzing signals within a passband of 300 Hz−3.5 kHz. As early as 4 weeks into the maturation process, spontaneous electrical activity emerged, characterized mainly by asynchronous spikes and bursts. As maturation progressed, neuronal network activity became more pronounced, with increased firing and burst rates by the 5th week. After 6 weeks, while neuronal activity remained detectable, a decrease in both single spike and burst firing rates was observed, along with less frequent network activity.

## 4 Discussion

The purity of iSN cultures is often limited by co-existent non-iSN cells which hinder reliable data interpretation and reproducibility. ISNs exhibit specialized functions, including nociception, thermoception, and mechanosensation. However, the presence of non-iSN cells in the culture can introduce signals unrelated to iSN-specific functions, potentially obscuring or diluting the functional responses of iSNs. This can result in inconsistent outcomes in assays designed to study iSN activity. Moreover, iSNs possess unique electrophysiological properties, such as distinct action potential firing patterns, which are essential for their function (Zurek et al., 2024). The inclusion of non-iSN cells can therefore complicate the interpretation of electrophysiological and molecular data (Pelkonen et al., 2021), making it challenging to attribute observed signatures specifically to iSNs (Schwartzentruber et al., 2018). Such heterogeneity may hinder efforts to accurately identify cellular mechanisms or pathways pertinent to sensory neurons.

In our studies, iSN cultures demonstrated a baseline purity of 60–70% without additional treatment, a result consistent with existing literature (Schwartzentruber et al., 2018). For context, other studies employing alternative differentiation protocols have reported iSN purity rates exceeding 95% under optimized conditions (Schrenk-Siemens et al., 2022; Kalia et al., 2024). This comparison underscores the variability in purity across methodologies and emphasizes the need to refine protocols to achieve an optimal balance between purity and cell viability.

Although several methods have been developed to reduce cellular heterogeneity in neuronal cultures of various origins (Hilgenberg and Smith, 2007; Thirumangalakudi et al., 2009; Irobi et al., 2010; Liu et al., 2013; Schwieger et al., 2016; Clark et al., 2017, 2021; Hirano et al., 2021), no standardized protocol is available (Lampert et al., 2020; Kalia et al., 2024). We have worked on improving the cellular purity of iSNs using a published protocol (Chambers et al., 2012) and report on the efficacy of cell incubation with 10 μM FdU for 24 h on day +10 split.

The first approach via cell split during differentiation of iSNs (Clark et al., 2017) did not increase iSN-to-total-cell count ratios. However, the total number of iSNs generated by differentiation including an additional split was substantially higher than our ctrl. Hence, early passaging during the differentiation process may ameliorate cell proliferation rates by augmenting the available growth area but does not influence the number of generated iSNs in relation to generated non-iSNs.

We could not reproduce the positive effect of MACS twice during the differentiation process increasing culture purity substantially (Hirano et al., 2021). In our hands, MACS did not improve the results compared to 10 μM FdU for 24 h. In addition, MACS induces cellular stress, and the protocol is time-consuming. A single MACS step led to an increased and more homogeneous non-iSN layer in some cases. This might be caused by using neural crest beads for selection, which are not only selective for iSNs but also for other neuronal cell types. Thus, we may have generated a more homogeneous culture of other neural crest-derived cells without increasing the number of iSNs.

While AraC is often used for animal-derived neuronal cultures (Batista Lobo et al., 2007; Schwieger et al., 2016), it was not feasible in our study. iSNs incubated with AraC either showed neuronal blebbing as a sign of stress, detached more frequently, or failed to survive maturation altogether. This may be due to the neurotoxic effects of AraC (Baker et al., 1991). AraC also induces apoptosis (Han et al., 2016) and reduces mitochondrial DNA in mouse DRG neurons (Zhuo et al., 2018). Low-dose AraC did not affect iSNs but also allowed the growth of non-iSNs. In comparison with FdU, AraC was not as efficient. The advantage of FdU over AraC was already shown in hippocampal cell cultures from postnatal rats, where both antimitotic agents were applied to reduce astrocyte contamination (Lesslich et al., 2022). Furthermore, it was suggested that AraC affects the location of transient receptor potential vanilloid 1 (TRPV1) in human DRG, which should be considered when working with iSN models (Anand et al., 2008). If FdU treatment is not sufficient for downstream experiments and a higher purification is needed, we recommend an additional replating step 25 min after the initial split. Replating at later time points was less effective as most cells had probably already been attached.

Treatment with 0.5 μM AraC shows more viable cells than the ctrl. High concentrations of FdU (10 and 20 μM) for extended treatment times resulted in marked iSN death. Similarly, AraC induced cell death at 5 and 10 μM, while lower concentrations (1 μM) were less detrimental, although the overall neuron-to-cell ratio still declined. This suggests that while AraC can be less toxic at lower concentrations, its overall impact on the cellular composition in culture remains important. The observed increase in cell viability compared to the ctrl in certain treatment groups likely does not fully reflect iSN health, but rather the survival of non-iSN cells at lower concentrations.

Eradication of non-iSN cells was not possible. Furthermore, we observed a decline in neuronal quality and elevated detachment rates in cultures that were almost free of non-iSNs. Thus, a certain proportion of non-iSNs might be beneficial for neuronal adhesion and providing nutrients to iSNs. A key challenge in cell isolation and purification protocols lies in the delicate balance between achieving high purity and maintaining cell viability. While high purity is often sought to reduce background noise and enhance experimental precision, many purification methods, especially those involving harsh conditions or extended processing, can compromise cell viability (Bowles et al., 2019; Saito-Diaz et al., 2023). This balance becomes particularly crucial in experiments requiring functional assays or subsequent cultivation. For many applications, it is essential to weigh purity against viability. For instance, genetic analysis, such as RNA sequencing or single-cell profiling, demands a high-purity cell population, but some reduction in viability can be acceptable as long as enough viable cells remain to generate meaningful data (Armand et al., 2021). In contrast, experiments involving functional assays, such as drug screening or multi-electrode arrays, often prioritize cell viability over absolute purity to ensure sufficient biological function is retained. ISNs generated following our optimized protocol expressed the same sensory and peripheral neuron markers Nav1.8 and PRPH, respectively, as iSNs generated using other protocols (Blanchard et al., 2015; Wainger et al., 2015). Furthermore, we could prove the expression of neuronal marker TUJ1. Gene expression levels of respective markers were confirmed in a previous study (Klein et al., 2024).

We successfully observed sustained electrical activity over a 6-week period, confirming the functionality of our iSNs. These findings indicate not only the successful differentiation of functional neurons but also their ongoing maturation. This prolonged activity likely reflects continuous synaptic and network refinements essential for the development of fully functional neuronal networks. However, to gain deeper insights into the maturation process and the long-term viability of functional iSNs, further experimentation will be required. While we assessed many different options to obtain purer iSN cultures, our study has some limitations. First, all experiments were performed using one iPSC line (*n* = 1 clone). Although inter-individual variation might influence the outcome (Volpato and Webber, 2020), we suggest that the general effects found within this study are transferable to other cell lines. In addition, we did not consider how additional modifications in the protocol, for example, the time point of addition and concentration of different small molecules, might affect the results (Lampert et al., 2020). The replating was only tested for attachment on glass coverslips coated with bMg; therefore, the results and optimal time points may differ when using plastic dishes and other coatings as these can affect the polarity, arborization, and maturation of cells (Nerli et al., 2020; Setien et al., 2020; Stil et al., 2023).

Nonetheless, our study offers a promising strategy for improving iSN purity without compromising cell viability or neuronal quality. However, further research considering inter-individual variability is warranted.

## Data Availability

The original contributions presented in the study are included in the article/supplementary material, further inquiries can be directed to the corresponding author.
